# Safety and Modulatory Effects of Humanized Galacto-Oligosaccharides on the Gut Microbiome

**DOI:** 10.3389/fnut.2021.640100

**Published:** 2021-04-07

**Authors:** Jason W. Arnold, Hunter D. Whittington, Suzanne F. Dagher, Jeffery Roach, M. Andrea Azcarate-Peril, Jose M. Bruno-Barcena

**Affiliations:** ^1^Division of Gastroenterology and Hepatology, Department of Medicine, School of Medicine, University of North Carolina, Chapel Hill, NC, United States; ^2^UNC Microbiome Core, Center for Gastrointestinal Biology and Disease, School of Medicine, University of North Carolina, Chapel Hill, NC, United States; ^3^Department of Plant and Microbial Biology, North Carolina State University, Raleigh, NC, United States; ^4^UNC Information Technology Services and Research Computing, University of North Carolina, Chapel Hill, NC, United States

**Keywords:** galactooligosaccharide (GOS), N-Acetyl-D-lactosamine (LacNAc), safety, human milk oligosaccharides (HMOs), LacNAc synthesis, mouse models

## Abstract

Complex dietary carbohydrate structures including β(1–4) galacto-oligosaccharides (GOS) are resistant to digestion in the upper gastrointestinal (GI) tract and arrive intact to the colon where they benefit the host by selectively stimulating microbial growth. Studies have reported the beneficial impact of GOS (alone or in combination with other prebiotics) by serving as metabolic substrates for modulating the assembly of the infant gut microbiome while reducing GI infections. N-Acetyl-D-lactosamine (LacNAc, Galβ1,4GlcNAc) is found in breast milk as a free disaccharide. This compound is also found as a component of human milk oligosaccharides (HMOs), which have repeating and variably branched lactose and/or LacNAc units, often attached to sialic acid and fucose monosaccharides. Human glycosyl-hydrolases do not degrade most HMOs, indicating that these structures have evolved as natural prebiotics to drive the proper assembly of the infant healthy gut microbiota. Here, we sought to develop a novel enzymatic method for generating LacNAc-enriched GOS, which we refer to as humanized GOS (hGOS). We showed that the membrane-bound β-hexosyl transferase (rBHT) from *Hamamotoa (Sporobolomyces) singularis* was able to generate GOS and hGOS from lactose and N-Acetyl-glucosamine (GlcNAc). The enzyme catalyzed the regio-selective, repeated addition of galactose from lactose to GlcNAc forming the β-galactosyl linkage at the 4-position of the GlcNAc and at the 1-position of D-galactose generating, in addition to GOS, LacNAc, and Galactosyl-LacNAc trisaccharides which were produced by two sequential transgalactosylations. Humanized GOS is chemically distinct from HMOs, and its effects *in vivo* have yet to be determined. Thus, we evaluated its safety and demonstrated the prebiotic's ability to modulate the gut microbiome in 6-week-old C57BL/6J mice. Longitudinal analysis of gut microbiome composition of stool samples collected from mice fed a diet containing hGOS for 5 weeks showed a transient reduction in alpha diversity. Differences in microbiome community composition mostly within the *Firmicutes* phylum were observed between hGOS and GOS, compared to control-fed animals. In sum, our study demonstrated the biological synthesis of hGOS, and signaled its safety and ability to modulate the gut microbiome *in vivo*, promoting the growth of beneficial microorganisms, including *Bifidobacterium* and *Akkermansia*.

## Introduction

Gut microbial communities play a critical role in the maintenance of host health (1, 2). Hence, beneficial modulation with probiotics (live microorganisms that when administered in adequate amounts provide a benefit to their hosts) (3) and prebiotics (selectively fermented dietary carbohydrate structures that promote the growth of beneficial microorganisms) (4, 5) is desirable and potentially effective translational therapeutics to treat gastrointestinal (GI) diseases linked to disrupted microbial communities (4, 6–9) [reviewed in (10)]. Synbiotics (combinations of prebiotics and probiotics) are also emerging as a focal point of GI biology research, as each component, individually and synergistically, could provide unique benefits reestablishing community resilience and host physiology (11, 12). In previous studies we evaluated highly pure β(1–4) galacto-oligosaccharides (GOS) formulations produced by the optimized version of the hexosyl-transferase gene from *Hamamotoa (Sporobolomyces) singularis* heterologously expressed in *Komagataella* (*Pichia*) *pastoris* (13, 14). This enzyme is one of the most promising catalysts in the field of glycobiology due to its high stability, highly desirable enzymatic properties, and the metabolism of its reaction products (GOS) by specific members of the gut microbial community, impacting its composition and function (15, 16). Beneficial members of the gut microbiota, including *Lactobacillus* and *Bifidobacterium*, hydrolyze GOS via β-galactosidases (17). *Lactobacillus rhamnosus* utilize PTS transporters to internalize GOS prior to hydrolysis (17), while other organisms like specific strains of *Bifidobacterium* (*bifidum*) secrete glycosyl hydrolases to break down complex carbohydrates, internalizing the products of hydrolysis (18). Short-chain fatty acids (SCFAs) generated as the result of GOS assimilation include acetate and lactate (17), which community members, including *Roseburia* and *Faecalibacterium*, can transform into butyrate (6, 19).

LacNAc is an essential component of human milk oligosaccharides (HMOs) and has been demonstrated to be a major bifidogenic factor in the 1950s (20–22). Several HMOs contain lactose (Galβ1-4Glc) at their reducing end, which can be elongated by the addition of β1-3- or β1-6-linked lacto-N-biose (Galβ1-3GlcNAc) or LacNAc (Galβ1-4GlcNAc). Lactose or the oligosaccharide can be then fucosylated by fucosyltransferases in α1-2, α1-3, or α1-4 linkage and/or sialylated by sialyltransferases in α2-3 or α2-6 linkage to yield a variety of terminal structures (23). The study by Yoshida et al. (24) characterized β-galactosidases of *Bifidobacterium longum* subsp. *infantis* to determine how this organism degrades type-1 (lacto-N-biose,) and type-2 (LacNAc,) isomers of HMOs. LacNAc has also been recognized as a building block of glycoproteins and glycolipids in the GI tract. These backbones serve to connect the core structure, which is directly linked to a protein or lipid aglycon with terminal sugars [reviewed in (25)]. LacNAc building blocks and terminal sugars also act as an important precursor of several blood group epitopes (Lewis A, Lewis B, sialyl Lewis A), which are involved in biological processes including fertilization (26), mediation of cell adhesion and pathogen adhesion to colonocytes (27–29).

Chemical and enzymatic synthesis processes have been the most frequently evaluated methods for LacNAc production (30). In recent years, glycoside hydrolases (EC 3.2.1.-) and β-galactosidases (EC 2.1.23) with both hydrolytic and transglycosylation activities, have gained special attention for regio- and stereo-selective synthesis of LacNAc oligosaccharides (https://www.cazypedia.org/index.php/Transglycosylases) (31–37). Enzymatic biosynthesis is considered the most efficient method for producing LacNAc due to specificity, synthesis in one-step reactions, low-cost substrates, sustainability, and overall low environmental impact, [reviewed in (38)]. Conversely, chemical methods to generate LacNAc require multiple reactive hydroxyl groups and laborious protocols for group protection and deprotection to control the stero- and regio-specificities (39, 40). Compared to enzymatic synthesis, generation of LacNAc by chemical synthesis has low yields, a cost-competitive disadvantage for industrial production, hindering the use of LacNAc as an additive in food products (30, 41, 42).

In this study, we describe a novel biological synthesis solution to produce N-Acetyl-lactosamine (LacNAc)-enriched GOS (which we refer to as humanized GOS, hGOS) using optimized *Hamamotoa (Sporobolomyces) singularis* β-hexosyl transferase [rBHT (13, 14)]. The enzyme generates LacNAc-enriched GOS as the product of the reaction between lactose as a galactose donor and N-Acetylglucosamine as acceptor. We first evaluated the efficiency of a *Komagataella* (*Pichia*) *pastoris* cell line carrying membrane-bound β-hexosyl transferase on the generation of GOS and hGOS from lactose and N-Acetyl-glucosamine. Then, conventionally-raised 6-week-old C57BL/6J mice were fed a control diet or modified diets containing GOS or hGOS for 14 days to evaluate its safety and impact on fecal microbial diversity and composition.

## Materials and Methods

### Generation of Dietary Carbohydrate Structures/Prebiotics GOS and hGOS

Membrane-bound β-hexosyl transferase from *Hamamotoa (Sporobolomyces) singularis* in *Komagataella* (*Pichia*) *pastoris* was produced as previously described (13, 14). The standard transgalactosylation reaction utilizing *Komagataella* (*Pichia*) *pastoris* resting cells (harboring membrane-bound enzyme) was initiated by adding standardized amounts of enzyme (1 U g^−1^ lactose) in 5 mM sodium phosphate buffer (pH 5.0) to a similarly buffered solution containing lactose (200 g liter^−1^) and N-Acetylglucosamine (25 g liter^−1^) at 30°C. Reaction products and substrates were analyzed by high-performance liquid chromatography (HPLC) (Shimadzu Corporation, Kyoto, Japan) under isocratic conditions at 65°C and a 0.5-ml min^−1^ flow rate. The mobile phase was water, and separation was performed by two columns in tandem a Supelco gel Ca++ (Supelco, PA), and HPX-42A (Bio-Rad, CA) columns (300 mm by 7.8 mm) coupled to an SPD-20MA and ELSD-LT II detectors (Shimadzu Corporation, Kyoto, Japan). The column was calibrated using galactosyl-lactose (Carbosynth, Berkshire, United Kingdom), LacNAc, Lactose, N-Acetylglucosamine, Glucose, and Galactose (Sigma-Aldrich, St. Louis, MO). Enzymatic activity was determined using 4-nitrophenyl β-D- glucopyranoside or oNP-Glc as substrate as per the previously described methods (13, 14).

### Human Equivalent Dose Calculation

The human equivalent dose (HED) for LacNAc was calculated for the animal study using the methods highlighted by the United States Food and Drug Administration and is based on the approximate body weight of the subject (43). The equation used is as follows:

$$\begin{array}{l} HED\;\left(\frac{mg}{kg}\right)=Animal\;Dose\;\left(\frac{mg}{kg}\right)\times \frac{Animal\;K_{m}}{Human\;K_{m}} \end{array}$$

Where the *K*_*m*_ factor is a number based on body surface area. For this study, we used an animal dose of 1,500 mg kg^−1^, based on ~30 mg LacNAc fed to a ~20 g mouse per day. Additionally, we used a *K*_*m*_ factor of 3 for mice and 16 to represent a 5 kg human infant (43). Using the formula above, the HED for this study represent an equivalent of 281.25 mg LacNAc per kg body weight in infants. While for a 20 kg child (*K*_*m*_= 25), the HED would be 180 mg kg^−1^.

### Animal Housing, Treatment, and Sample Collection

All animal studies were approved by the Institutional Animal Care and Use Committee (IACUC) of the University of North Carolina at Chapel Hill (Approved protocol number: 19-084).

A total of 50 6-week-old C57BL/6J mice were co-housed at random in groups of 5–6 animals and fed a defined diet (D17121301; Research Diets INC.) containing no prebiotics to normalize the gut microbiota for 2 weeks. After a 2-week standardization period, fresh stool samples were collected directly from the anus of each animal into a sterile tube. To avoid cage batch effects, animals were moved into paired housing such that no two animals from the same standardization group were co-housed. Each animal was considered one experimental unit. Upon reassigning housing (**Figure 2A**), animal pairs were split into three distinct groups, each of which began feeding on either the defined control diet (D17121301) (*n* = 17), GOS diet (D17121302) (*n* = 17) in which 71.8 g of cellulose per kilogram diet was replaced with 71.8 g of pure GOS, or hGOS (D18121401) (*n* = 16) where LacNAc represented a 1% (w/w). Composition of each diet is detailed in Supplementary Table 1. Each diet was offered *ad libitum* for 2 weeks prior to stool sample collection. Individual animal mass and dietary consumption were measured daily during the first 14 days of the dietary study to assess animal growth and food consumption (total food consumed in a cage/2 = individual animal food consumption) between diet groups. After day-14 sample collection, half of the animals in each treatment group were removed for a tangential study and all remaining animals (total=24, *n* = 8 per diet) continued to consume their respective diets *ad libitum*, with stool sample collections occurring at three additional time points each ~1-week apart prior to animal sacrifice. At the conclusion of the trial on day 38, each animal was euthanized via CO_2_ asphyxiation and cervical dislocation.

### Nucleic Acid Isolation

Total DNA was extracted from fecal pellets using the Qiagen ClearMag Extraction system on KingFisher Flex Magnetic Bead processing instrument as described (15). Briefly, stool samples were transferred to a screwcap tube containing 10 mg of sterile acid-washed glass beads (0.1–0.5 mm diameter) and 700 μl PM1 solution (Qiagen, Valencia, CA). Samples were homogenized for 5-min at 15 Hz in Qiagen Tissue Lyser II (Qiagen). Bead-beaten samples were treated with IRS-PCR inhibitor remover solution (Qiagen) (3:1; lysate:IRS ratio) overnight at 4°C and transferred to KingFisher Deep-well plate containing ClearMag magnetic beads and binding buffer (Qiagen). Sample plates were subsequently processed on KingFisher Flex instrument to isolate and wash DNA. DNA was quantified with Quant-iT™ PicoGreen® dsDNA reagent (Molecular Probes, Thermo Fisher Scientific, Waltham, MA) and stored at −20°C.

### 16S rRNA Amplicon Sequencing

Total DNA was subject to amplification of the V4 variable region of the 16S rRNA gene using primers 515F and 806R (44) with Illumina adaptors. Amplicons were barcoded using Illumina dual-index barcodes [Index 1(i7) and Index 2(i5)], purified using Agencourt® AMPure® XP reagent (Beckman Coulter, Brea, CA) and quantified with Quant-iT™ PicoGreen™ dsDNA Reagent (Molecular Probes, Thermo Fisher Scientific). Libraries were pooled in equimolar amounts and sequenced on HiSeq2500 instrument (Illumina, San Diego, CA).

### Sequencing Data Analysis

Analysis of 16S rRNA amplicon sequencing data was carried out using the QIIME2 pipeline as described (45). Briefly, sequences were grouped into Operational Taxonomic Units (OTUs) using UCLUST (46). OTU sequences were aligned, and phylogenetic trees were built (47). Before generating the phylogenetic tree, the overall OTU table was collapsed using the “taxa collapse” plugin in QIIME2. The set of representative sequences was then trimmed to include only one representative sequence for each collapsed OTU. The filtered set of representative sequences was then aligned using MAFFT, and a phylogenetic tree was generated from the alignment using RAxML (48, 49). The phylogenetic tree was finally annotated with presence/absence data using iToL and PhyloToAST (50–52). Alpha and beta diversity metrics were calculated in R 4.0.3 using the phyloseq and vegan packages (53–55). Only data from young animals (6 weeks old) were used in the calculation of diversity metrics. Both the Shannon entropy and inverse Simpson indexes were calculated to ensure an accurate estimation of the true alpha diversity of the samples. Beta diversity was calculated using principal coordinate analysis (PCoA) of the weighted UniFrac distances (56).

### Statistical Analysis

Data were evaluated for homogeneity of variance using Levene's test. Statistical significance of alpha diversity was evaluated using a repeated-measures ANOVA followed by Tukey's Honest Significant Difference test to separate means. The 95% confidence ellipses for beta diversity plots were calculated in R 4.0.3 using ggplot2 (57). Beta diversity statistical analyses were performed using the PERMANOVA and PERMDISP functions of the vegan package in R 4.0.3. All statistical analysis results for the alpha and beta diversity analyses can be found in Supplementary Table 2. The α for all statistical tests was fixed at 0.05.

### Availability of Data and Materials

All sequencing data has been submitted to the NCBI repository and can be accessed via the following accession number: PRJNA681811.

## Results

### rBHT Catalyzed the Repeated Addition of Galactose From Lactose to N-Acetylglucosamine

**Figure 1:**
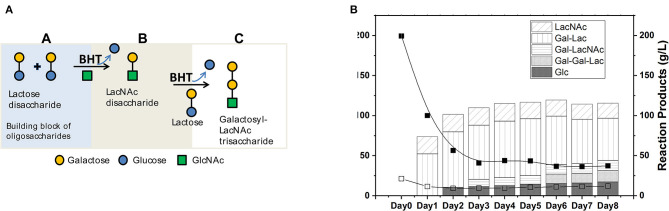
**(A)** Illustrative representation of catalysis of hGOS from carbohydrate constituents. **(B)** Time course evolution in g/L of dietary carbohydrate structures generation and residual substrates in g/L over 8 days; Lactose (Lac), N-Acetylglucosamine (GlcNAc), and Glucose (Glc). The products of the reaction were N-Acetyl-lactosamine (LacNAc), Galactosyl-β(1–4)lactose (Gal-Lac), Galactosyl-β(1–4)N-Acetyl-lactosamine (Gal-LacNAc), and Galactosyl-β(1–4)Galactosyl-β(1–4)lactose (Gal-Gal-Lac). The reactions were performed using whole cells membrane-bound protein (1 U rBHT.g^−1^ lactose). The initial conditions of the reactions contained 200 g/L lactose; 25 g/L N-Acetylglucosamine (GlcNAc), in 5 mM sodium phosphate buffer (pH 5.0) and incubated at 30°C. Samples were removed periodically and separated by HPLC and quantified with sequential ELSD and PDA detectors.

The reactions catalyzed by the rBHT enzyme were regio-selective, forming the β-galactosyl linkage at the 4-position of the GlcNAc and the 1-position of D-galactose, synthesizing various glycoconjugates directly from soluble GlcNAc. The obtained products, in addition to GOS, included Gal-β(1, 4)GlcNAc (LacNAc, Figure 1A, panel B) disaccharides and Galβ-(1, 4)Galβ-(1, 4)GlcNAc (Galactosyl-LacNAc, Figure 1A, panel C) trisaccharides, which were produced by two sequential transgalactosylations. Figure 1B shows the kinetics of the reaction performed during 8 days of incubation using rBHT polypeptides (e.g., whole cells displaying membrane-bound enzyme). The enrichment of GOS with LacNAc at a ratio lactose/N-Acetylglucosamine of 8:1 performed for these experiments (200 g/L lactose and 25 g/L GlcNAc) generated 25 g/L of LacNAc and 100 g/L hGOS after 48 h of incubation. At this time point, the reaction was terminated, and the products of the reaction (hGOS) were freeze-dried.

### Animal Health and Diet Consumption

**Figure 2:**
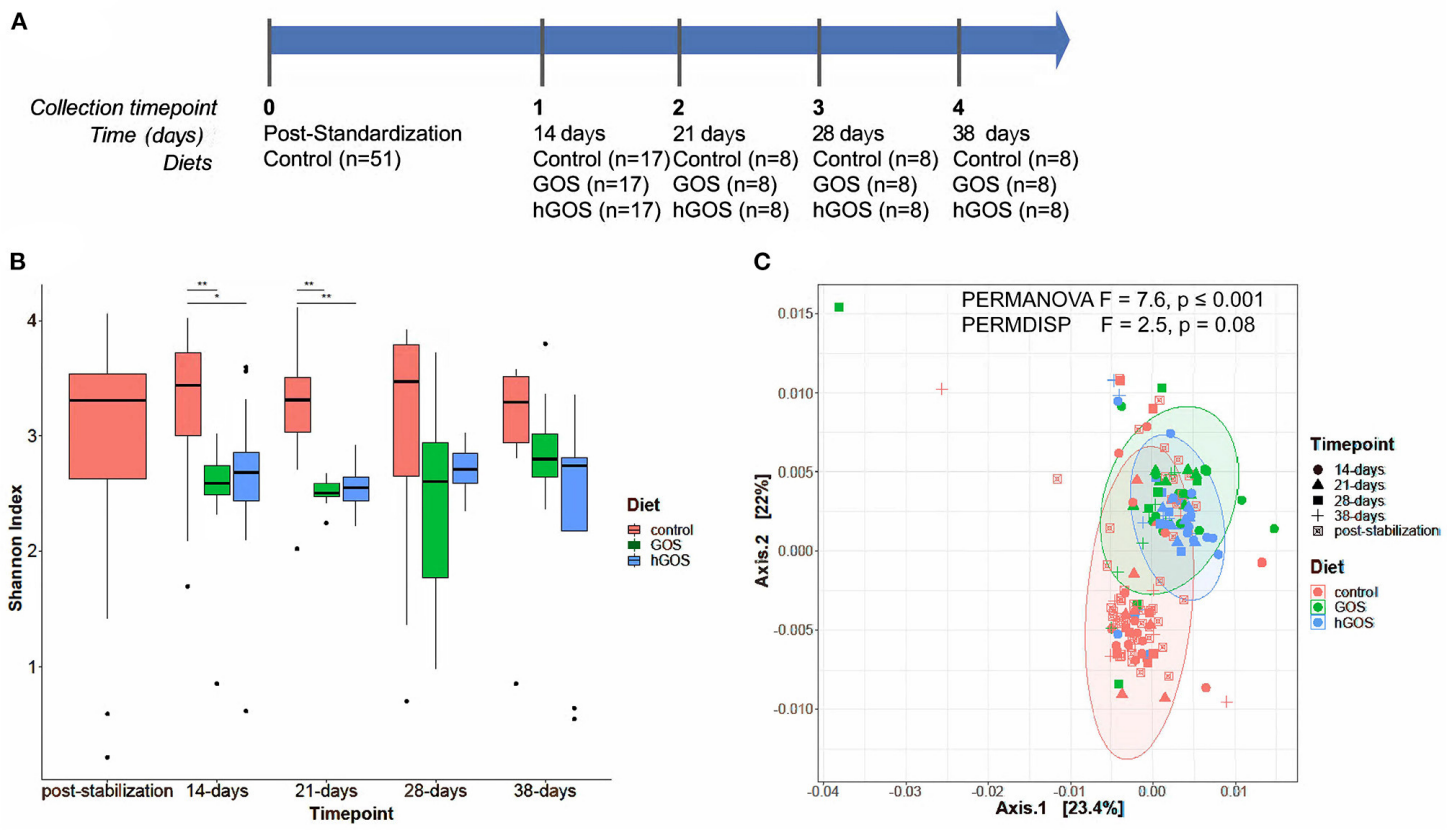
**(A)** Experimental timeline of the animal study, delineating the number of animals in each experimental group, and the duration between sample collection and analysis. **(B)** Box plots show changes in Shannon diversity values between groups fed control (red), GOS (green), or hGOS (blue) diets over time. Significant differences in Shannon diversity between diets are indicated by bars and asterisks, with *indicating *p* < 0.05 and **indicating *p* < 0.01. Only statistically significant differences are shown. **(C)** PCoA, PERMANOVA, and PERMDISP analyses of samples between time points show significant differences in clustering as a function of the diet.

We conducted an animal experiment with conventional 6-week-old C57BL/6J mice fed a control diet, or modified diets containing GOS or hGOS to assess their impact on the gut microbiome (Figure 2A). Results showed no impact of GOS or hGOS diets on weight or daily food consumption. Each mouse consumed ~3 g of food per day, with no significant differences between the diets (Supplementary Figure 1). Therefore, based on the formulation of each prebiotic diet, we calculated that the dose of prebiotic consumed per day by each mouse was 0 mg/day (on control diet), and ~190 mg/day of total prebiotic (GOS, hGOS). This estimate translates to ~171 mg/day of GOS (GOS diet) and ~30 mg/day of LacNAc (hGOS diet) based on prebiotic formulation data.

### Modulation of the Gut Microbiota by hGOS

After 2 weeks of feeding on diets containing prebiotics, animals exhibited a significant (repeated measures ANOVA *p* < 0.05) reduction in alpha diversity (Figure 2B). Over the length of the study, diversity of prebiotic-fed animals returned to values comparable to the control diet with no statistically significant differences between groups at day 28. PCoA plots revealed distinct clustering of hGOS-fed animals, which displayed a much tighter dispersion pattern compared to control animals (PERMDISP *p* = 0.009), suggesting a higher similarity between communities within hGOS-fed than control-fed animals (Figure 2C). Spatial medians were significantly different between groups (PERMANOVA *F* = 7.6463, *p* ≤ 0.001).

**Figure 3:**
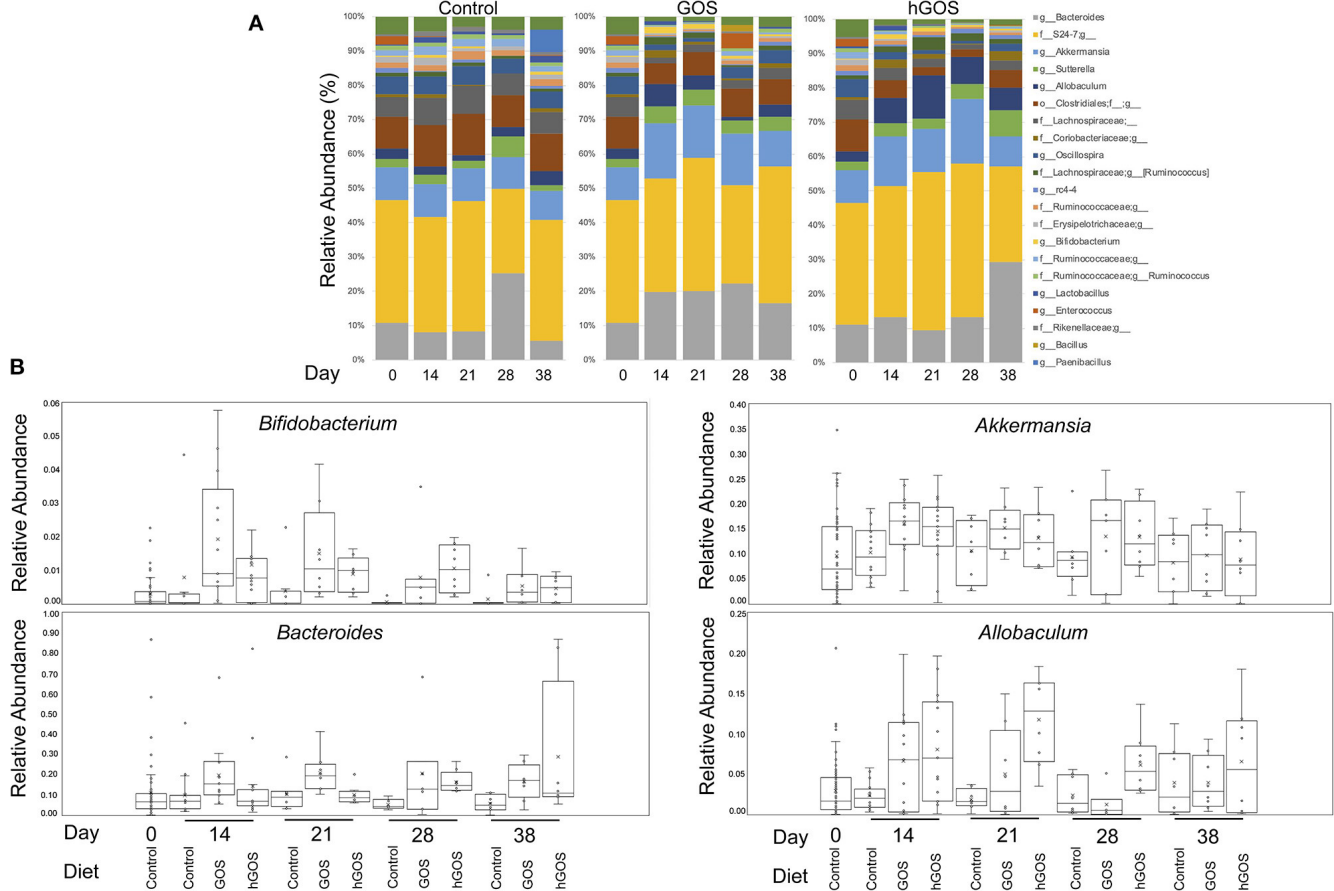
**(A)** Genus-level taxonomy plots reveal highly abundant taxa in each group at each time point in the study. Changes associated with diet were observed immediately (day 14) and were persistent throughout the trial. **(B)** *Bifidobacterium, Akkermansia, Bacteroides*, and *Allobaculum* were significantly increased in prebiotic-fed animals at 14 days compared to controls.

Taxonomy plots of relative microbial abundance revealed the genus-level variability between animals fed control diets, and those consuming either GOS or hGOS diets over time (Figure 3A). The most dramatic changes in the assembled microbial communities were observed between timepoint 0 and 14 days after introducing prebiotic diets. Changes between the communities within prebiotic-fed animals after 14 days were minimal. Analysis of Composition of Microbiome (ANCOM) used to further explore microbial abundance changes within the communities of prebiotic-fed animals across all time points revealed an increased relative abundance of beneficial microorganisms including *Akkermansia, Bifidobacterium*, and *Bacteroides*, along with *Allobaculum* in both GOS and hGOS diets. The dietary interventions reduced the relative abundance of *Butyricicoccus, Clostridium, Turicibacter*, and *Lachnospiraceae* across all time points (Figures 3A,B, Supplementary Figure 2).

**Figure 4:**
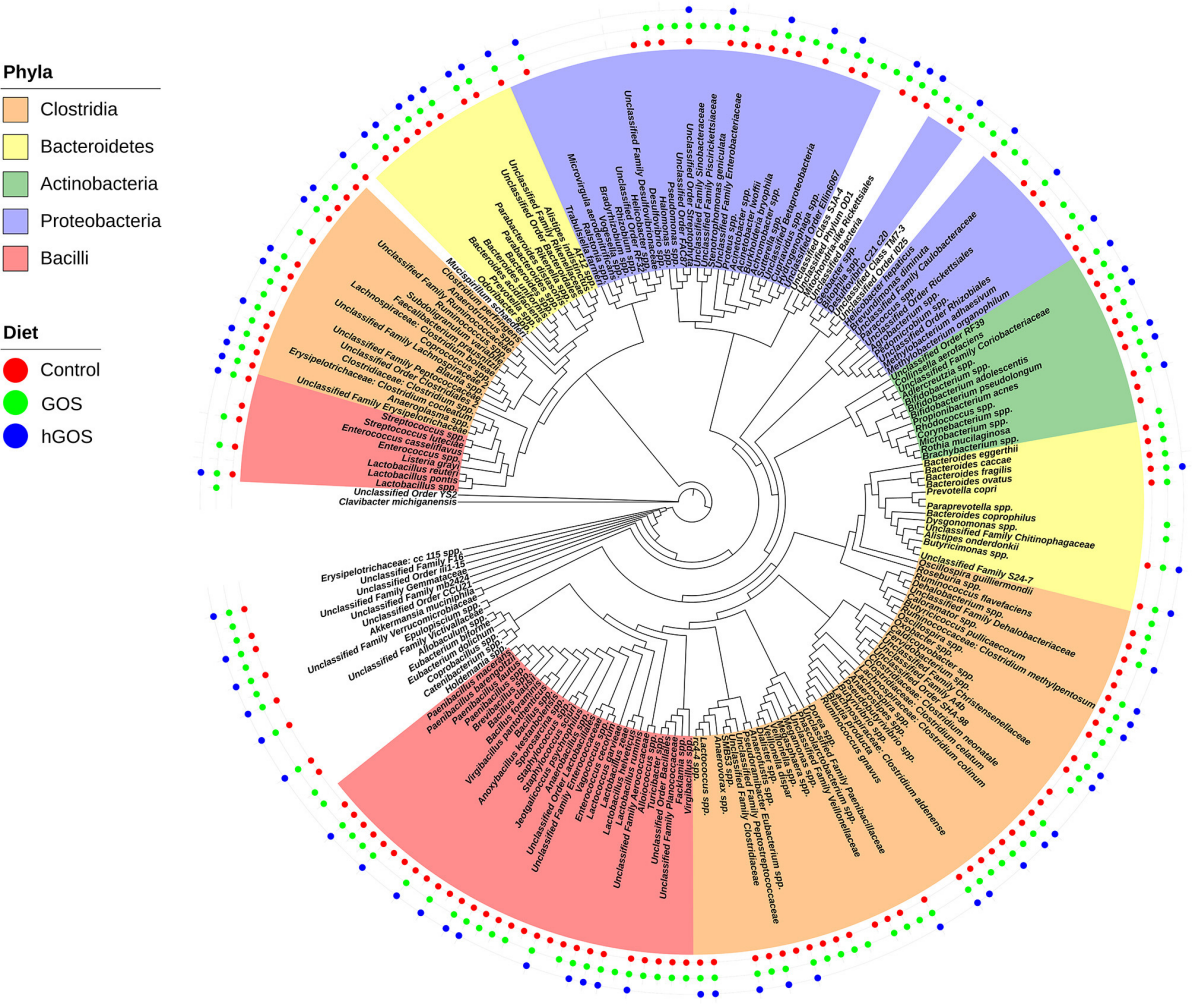
Phylogenetic tree shows the taxa that are present or absent in animals fed each experimental or control diet. Taxa that are highlighted by phyla and presence is designated by adding a colored dot for each diet in which the taxa were observed.

Figure 4 shows a phylogenetic tree generated using PhyloToAST that includes 117 unique OTUs detected in at least one of the three diets examined. Of the 117 unique OTUs detected, 76 OTUs were detected in all conditions (Control, GOS, hGOS), and 40 were detected in control and GOS but not in hGOS fed mice, while none was detected in control and hGOS but not GOS fed mice. The majority of the OTUs detected in the control and GOS mice, but not in hGOS mice, belonged to the *Firmicutes* phylum. Taxa not detected in the hGOS group compared to control and GOS group within the Phylum *Firmicutes* and Class *Bacilli* included *Lactobacillus reuteri, L. zeae*, species of *Enterococcus, Brevibacillus, Paenibacillus, Anaerobacillus, Virgibacillus, Facklamia, Unclassified Lactobacillales, Bacillales, Enterococcaceae*, and *Planococcaceae*. Within the Phylum *Firmicutes* and Class *Clostridia*, the following were not detected in the hGOS group: *Veillonella dispar, Ruminococcus flavefaciens, Faecalibacterium prausnitzi*, species of *Butyrivibrio, Pseudobutyrivibrio, Lachnospira, Oxobacter, Roseburia, Dialister, Veillonella, Phascolarctobacterium, Anaerotruncus, Blautia* and Unclassified *Clostridiales, Clostridiaceae*, and *Veillonellaceae*. Only 3 OTUs corresponding to the Phylum *Bacteroidetes* (*Bacteroides eggerthii, B. caccae, and B. fragilis*) and 7 Proteobacteria (*Burkholderia bryophila, Acinetobacter, Bilophila, Hydrogenophaga, Pseudomonas, Halomonas*, and Unclassified *Enterobacteriaceae*) were not detected in the hGOS group. Supplementary Table 1 presents the mean relative abundance values for each observed OTU in each of the experimental conditions.

## Discussion

Prebiotics, including GOS, are selectively fermented by gut microorganisms and promote the growth of beneficial microorganisms when consumed in adequate amounts (16, 17). In this study, we report the biological synthesis of hGOS enriched in LacNAc and determined its lack of adverse effects by determining the impact of feeding on the gut microbiome of healthy 6-week-old C57BL/6J mice in comparison with defined control and GOS-containing diets.

Traditionally, higher values of gut microbiota diversity has been associated with good health (58–60). In our study, feeding of both GOS and hGOS-enriched diets initially reduced diversity, even when constituents of the gut microbial community considered beneficial (*Bifidobacterium, Akkermansia*, and species of *Bacteroides*) increased. Diversity increased at 28 and 38 days, suggesting that sustained hGOS feeding would lead to a diversity comparable to the control group. In addition, we did not observe differences in dietary consumption or weight in prebiotic-fed animals compared to the control group. We have recently reported an initial decreased diversity in 6- and 60-week old GOS-fed C57BL/6J mice after 2 weeks (15), which is in accordance with studies of GOS-supplemented infant formula (61) but contrast with other studies on human adults (6, 62) and young or adult BALB/c mice that showed no changes on diversity due to GOS feeding (63, 64). Considering the biochemical structure of GOS and hGOS and their similarity to HMOs, it makes sense that these prebiotics exert a restrictive selection of microorganisms to only microbes capable of establishing a mutualistic relationship with the host as observed in breastfed infants (65). The restrictive colonization effect leads in babies to the successive establishment of different bacterial groups, from aerotolerant bacteria to progressively stricter anaerobes (66, 67), and could provide in adults and older adults a strategy to beneficially modulate the gut microbiome by the subsequent introduction of microbial network units (68).

Members of the gut microbial community including strains of *Bifidobacterium* and *Lactobacillus* encode galactosidases genes that hydrolyze complex carbohydrates including GOS, as demonstrated in our previous and current studies (15–17, 19, 69) generating products which other members of the gut microbiota can further utilize through cross-feeding (19, 70). Due to structural similarities between the dietary carbohydrate structures contained in GOS and hGOS, it can be expected that their hydrolysis will result in similar molecules, including lactate and acetate, which could subsequently be utilized to generate other SCFAs of biological relevance, including butyrate. We anticipated that the additional LacNAc residues in hGOS would provide an additional substrate for bacterial enzymatic systems, allowing for different microorganisms to utilize these compounds compared to GOS. However, our study was not able to detect bacterial groups that used hGOS but not GOS. Further experiments will be required to characterize the gut bacterial metabolism of hGOS. Among other changes in the gut microbiota, feeding GOS and hGOS increased the abundance of *Akkermansia muciniphila*, a microorganism that predominantly utilizes mucin as its energy source. GOS enrichment of *Akkermansia* is likely a consequence of increased mucin production (15). However, hGOS (containing LacNAc) may be utilized directly by *Akkermansia* due to a similar LacNAc structure found in hGOS and mucin (71). These findings are consistent with our previous animal studies (15, 16); however, animal models have significant limitations due to fundamental differences between human and mouse-originated microorganisms (72). Further studies are currently underway to better assess the impacts of hGOS on human bacterial isolates, with the ultimate goal of developing a prebiotic optimized for human consumption.

Finding the proper dose of a new therapeutic compound is vital not only to ensure safety and efficacy in clinical trials but is also necessary to ensure the economic feasibility of the new product. For GOS, a low dose (below 2 g per day) may not elicit the desired modulatory effect (6, 73), while an excessively high dose (over 15 grams per day) may induce undesired GI effects (6, 74). Studies have shown the importance of translating the dose of a compound validated in animal models to the HED [reviewed in (75)]. Here, we demonstrated the lack of adverse effects of a HMO mimetic composition, LacNAc-enriched GOS, and its ability to modulate the gut microbiome at a HED of 180 mg kg^−1^ day^−1^. The values tested during our experiments are in accordance with the recommended values by the EFSA panel (76) for 2′-O-fucosyllactose (2′-FL) and lacto-N-neotetraose (LNnT). The tested HED was six times higher than the calculated average amount of LacNAc consumed in a day by a 5 kg infant (1,400 vs. 232 mg day^−1^), potentially highlighting the lack of adverse effects of LacNAc, even at a higher-than-physiological doses (77, 78).

Breast milk is undoubtedly the optimal source of nutrition for the human infant (79) and, until recently, the HMOs present in mother's milk could not be replicated in enough quantities to add to infant formulas. Five years ago, the study by Marriage et al. (80) showed that weight, length, head circumference growth and uptake of 2′FL, measured in the blood and urine, were similar to those of breastfed babies and today, some infant formulas have already incorporated this HMO. As a major building block of HMOs, the addition of LacNAc to the existing list of prebiotic compounds is of paramount importance for the further development of safe, nutritionally, and immunologically complete formulas. Hence, our study represents the first step in evaluating the safety and efficacy of enzymatically produced hGOS in an animal model of weaned human infants.

## Data Availability Statement

All sequencing data has been submitted to NCBI repository and can be accessed via the following accession number: PRJNA681811.

## Ethics Statement

The animal study was reviewed and approved by Institutional Animal Care and Use Committee of the University of North Carolina at Chapel Hill (Approved protocol number: 19-084).

## Author Contributions

JA performed and analyzed animal experiments. HW and JR curated and analyzed the amplicon sequencing data presented in the manuscript. SD produced the humanized GOS for the animal experiments. MA-P and JB-B designed the experiments and edited the manuscript. All authors contributed to the writing of the manuscript.

## Conflict of Interest

The authors declare that the research was conducted in the absence of any commercial or financial relationships that could be construed as a potential conflict of interest.

## Supplementary Material

Supplementary Figure 1 Bodyweight and daily dietary consumption datasets are included to show that animal growth and intake were not inhibited by the introduction of either experimental diet (GOS and hGOS).

Supplementary Figure 2 Bacterial taxa shown to have a significantly reduced relative abundance in the presence of hGOS.

Supplementary Table 1 Composition of diets used in the animal study.

## References

[1] Arnold JW Roach J Azcarate-Peril MA. emerging technologies for gut microbiome research. Trends Microbiol. (2016) 24:887–901. 10.1016/j.tim.2016.06.008 27426971 PMC5074899

[2] Madupu R Szpakowski S Nelson K. Microbiome in human health and disease. Sci Prog. (2013) 96:153–70. 10.3184/003685013X13683759820813 23901633 PMC10365526

[3] Reid G Jass J Sebulsky MT McCormick JK. Potential uses of probiotics in clinical practice. Clin Microbiol Rev. (2003) 16:658–72. 10.1128/CMR.16.4.658-672.2003 14557292 PMC207122

[4] de Vrese M Schrezenmeir J. Probiotics, prebiotics, and synbiotics. Adv Biochem Eng Biotechnol. (2008) 111:1–66. 10.1007/10_2008_097 18461293

[5] Roberfroid M Gibson GR Hoyles L McCartney AL Rastall R Rowland I. Prebiotic effects: metabolic and health benefits. Br Nutr J. (2010) 104(Suppl 2):S1–63. 10.1017/S0007114510003363 20920376

[6] Azcarate-Peril MA Ritter AJ Savaiano D Monteagudo-Mera A Anderson C Magness ST. Impact of short-chain galactooligosaccharides on the gut microbiome of lactose-intolerant individuals. Proc Natl Acad Sci USA. (2017) 114:E367–75. 10.1073/pnas.1606722113 28049818 PMC5255593

[7] Duncan SH Flint HJ. Probiotics and prebiotics and health in ageing populations. Maturitas. (2013) 75:44–50. 10.1016/j.maturitas.2013.02.004 23489554

[8] Reid G Sanders ME Gaskins HR Gibson GR Mercenier A Rastall R. New scientific paradigms for probiotics and prebiotics. J Clin Gastroenterol. (2003) 37:105–18. 10.1097/00004836-200308000-00004 12869879

[9] Vitetta L Briskey D Alford H Hall S Coulson S. Probiotics, prebiotics and the gastrointestinal tract in health and disease. Inflammopharmacology. (2014) 22:135–54. 10.1007/s10787-014-0201-4 24633989

[10] Belorkar SA Gupta AK. Oligosaccharides: a boon from nature's desk. AMB Express. (2016) 6:82. 10.1186/s13568-016-0253-5 27699701 PMC5047869

[11] Markowiak P Slizewska K. Effects of probiotics, prebiotics, and synbiotics on human health. Nutrients. (2017) 9:1021. 10.3390/nu9091021 28914794 PMC5622781

[12] West NP Pyne DB Cripps A Christophersen CT Conlon MA Fricker PA. Gut balance, a synbiotic supplement, increases fecal *Lactobacillus paracasei* but has little effect on immunity in healthy physically active individuals. Gut Microbes. (2012) 3:221–7. 10.4161/gmic.19579 22572834 PMC3427214

[13] Dagher SF Azcarate-Peril MA Bruno-Barcena JM. Heterologous expression of a bioactive beta-hexosyltransferase, an enzyme producer of prebiotics, from Sporobolomyces singularis. Appl. Environ Microbiol. (2013) 79:1241–9. 10.1128/AEM.03491-12 23241974 PMC3568590

[14] Dagher SF Bruno-Bárcena JM. A novel N-terminal region of the membrane β-hexosyltransferase: its role in secretion of soluble protein by Pichia pastoris. Microbiology. (2016) 162:23–34. 10.1099/mic.0.000211 26552922 PMC5974927

[15] Arnold JW Roach J Fabela S Moorfield E Ding S Blue E. The pleiotropic effects of prebiotic galacto-oligosaccharides on the aging gut. Microbiome. (2021) 9:31. 10.1186/s40168-020-00980-0 33509277 PMC7845053

[16] Monteagudo-Mera A Arthur JC Jobin C Keku TO Bruno Barcena JM Azcarate-Peril MA. High purity galacto-oligosaccharides enhance specific Bifidobacterium species and their metabolic activity in the mouse gut microbiome. Benef Microb. (2016) 3:1–18. 10.3920/BM2015.0114 26839072 PMC4974821

[17] Arnold JW Simpson JB Roach J Bruno-Barcena JM Azcarate-Peril MA. Prebiotics for lactose intolerance: variability in galacto-oligosaccharide utilization by intestinal *Lactobacillus rhamnosus*. Nutrients. (2018) 10:1517. 10.3390/nu10101517 30332787 PMC6213946

[18] Møller PL Jørgensen F Hansen OC Madsen SM Stougaard P. Intra- and extracellular beta-galactosidases from *Bifidobacterium bifidum* and *B. infantis:* molecular cloning, heterologous expression, comparative characterization. Appl Environ Microbiol. (2001) 67:2276–83. 10.1128/AEM.67.5.2276-2283.2001 11319112 PMC92867

[19] Riviere A Selak M Lantin D Leroy F De Vuyst L. Bifidobacteria and butyrate-producing colon bacteria: importance and strategies for their stimulation in the human gut. Front Microbiol. (2016) 7:979. 10.3389/fmicb.2016.00979 27446020 PMC4923077

[20] György P. N-Containing saccharides in human milk. In: Wolstenholme GEW, O'Connor M, editors. Ciba Foundation Symposium - Chemistry and Biology of Mucopolysaccharides. J. and A. Churchill Ltd (1958). p. 140–56. 10.1002/9780470719060.ch9

[21] György P Norris RF Rose CS. Bifidus factor. *I*. A variant of *Lactobacillus bifidus* requiring a special growth factor. Arch Biochem Biophys. (1954) 48:193–201. 10.1016/0003-9861(54)90323-9 13125589

[22] György P Rose CS. Microbiological studies on growth factor for *L. bifidus var. pennsylvanicus*. Exp Biol Med. (1955) 90:219–23. 10.3181/00379727-90-21988 13273403

[23] Bode L. Recent advances on structure, metabolism, and function of human milk oligosaccharides. J Nutrit. (2006) 136:2127–30. 10.1093/jn/136.8.2127 16857829

[24] Yoshida E Sakurama H Kiyohara M Nakajima M Kitaoka M Ashida H. Bifidobacterium longum subsp. infantis uses two different beta-galactosidases for selectively degrading type-1 and type-2 human milk oligosaccharides. Glycobiology. (2012) 22:361–8. 10.1093/glycob/cwr116 21926104

[25] van den Eijnden DH. On the origin of oligosaccharide species—glycosyltransferases in action. In: Ernst B, Hart GW, Sinaý P, . Carbohydrates in Chemistry Biology. Weinheim: Wiley-VCH (2000). p. 589–624. 10.1002/9783527618255.ch23

[26] Kerr CL Hanna WF Shaper JH Wright WW. Lewis X-containing glycans are specific and potent competitive inhibitors of the binding of ZP3 to complementary sites on capacitated, acrosome-intact mouse sperm1. Biol Reprod. (2004) 71:770–7. 10.1095/biolreprod.103.023812 15128590

[27] Bode L. Human milk oligosaccharides: every baby needs a sugar mama. Glycobiology. (2012) 22:1147–62. 10.1093/glycob/cws074 22513036 PMC3406618

[28] Du J Yarema KJ. Carbohydrate engineered cells for regenerative medicine. Adv Drug Deliv Rev. (2010) 62:671–82. 10.1016/j.addr.2010.01.003 20117158 PMC3032398

[29] Lane JA Mehra RK Carrington SD Hickey RM. The food glycome: a source of protection against pathogen colonization in the gastrointestinal tract. Int J Food Microbiol. (2010) 142:1–13. 10.1016/j.ijfoodmicro.2010.05.027 20580113

[30] Krasnova L Wong C.-H. Oligosaccharide synthesis and translational innovation. J Am Chem Soc. (2019) 141:3735–54. 10.1021/jacs.8b11005 30716271 PMC6538563

[31] Arreola SL Intanon M Wongputtisin P Kosma P Haltrich D Nguyen TH. Transferase activity of Lactobacillal and Bifidobacterial β-Galactosidases with various sugars as galactosyl acceptors. J Agric Food Chem. (2016) 64:2604–11. 10.1021/acs.jafc.5b06009 26975338 PMC4819807

[32] Black BA Lee VSY Zhao YY Hu Y Curtis JM Gänzle MG. Structural identification of novel oligosaccharides produced by *Lactobacillus bulgaricus* and *Lactobacillus plantarum*. J Agric Food Chem. (2012) 60:4886–94. 10.1021/jf300917m 22497208

[33] Bridiau N Maugard T. A comparative study of the regioselectivity of the β-galactosidases from *Kluyveromyces lactis* and *Bacillus circulans* in the enzymatic synthesis of N-Acetyl-lactosamine in aqueous media. Biotechnol Prog. (2011) 27:386–94. 10.1002/btpr.542 21344676

[34] Fischöder T Laaf D Dey C Elling L. Enzymatic synthesis of N-Acetyllactosamine (LacNAc) Type 1 oligomers and characterization as multivalent galectin ligands. Molecules. (2017) 22:1320. 10.3390/molecules22081320 28796164 PMC6152129

[35] Li W Sun Y Ye H. Synthesis of oligosaccharides with lactose and N-acetylglucosamine as substrates by using β-D-galactosidase from *Bacillus circulans*. Eur Food Res Technol. (2010) 231:55–63. 10.1007/s00217-010-1254-2

[36] Lombard V Golaconda Ramulu H Drula E Coutinho PM Henrissat B. The carbohydrate-active enzymes database (CAZy) in 2013. Nucleic Acids Res. (2014) 42:D490–D5. 10.1093/nar/gkt1178 24270786 PMC3965031

[37] The CC. Ten years of CAZypedia: a living encyclopedia of carbohydrate-active enzymes. Glycobiology. (2018) 28:3–8. 10.1093/glycob/cwx089 29040563

[38] Karimi Alavijeh M Meyer AS Gras SL Kentish SE. Simulation and economic assessment of large-scale enzymatic N-acetyllactosamine manufacture. Biochem Eng J. (2020) 154:107459. 10.1016/j.bej.2019.107459

[39] Mong TKK Huang C-Y Wong C-H. A new reactivity-based one-pot synthesis of N-Acetyllactosamine oligomers. J Org Chem. (2003) 68:2135–42. 10.1021/jo0206420 12636372

[40] Romanò C Oscarson S. Synthesis of lactosamine-based building blocks on a practical scale and investigations of their assembly for the preparation of 19F-labelled LacNAc oligomers. Organ Biomol Chem. (2019) 17:2265–78. 10.1039/C8OB03066A 30724303

[41] Benkoulouche M Fauré R Remaud-Siméon M Moulis C André I. Harnessing glycoenzyme engineering for synthesis of bioactive oligosaccharides. J R Soc Interface Focus. (2019) 9:20180069. 10.1098/rsfs.2018.0069 30842872 PMC6388017

[42] Martins GN Ureta MM Tymczyszyn EE Castilho PC Gomez-Zavaglia A. Technological aspects of the production of fructo and galacto-oligosaccharides. enzymatic synthesis and hydrolysis. Front Nutrit. (2019) 6:78. 10.3389/fnut.2019.00078 31214595 PMC6554340

[43] U.S. Department of Health and Human Services Food and Drug Administration. (2005). Guidance for Industry: Estimating the Maximum Safe Starting Dose in Initial Clinical Trials for Therapeutics in Adult Healthy Volunteers. Rockville, MD: Center for Drug Evaluation and Research (CDER), 7.

[44] Khan F. Review of Soil Taxonomy Information for Submitted Environmental Fate Studies of Ametroctradin. Washington, DC: U.S. Environmental Protection Agency (2012).

[45] Allali I Arnold JW Roach J Cadenas MB Butz N Hassan HM. A comparison of sequencing platforms and bioinformatics pipelines for compositional analysis of the gut microbiome. BMC Microbiol. (2017) 17:194. 10.1186/s12866-017-1101-8 28903732 PMC5598039

[46] Edgar RC. Search and clustering orders of magnitude faster than BLAS. Bioinformatics. (2010) 26:2460–1. 10.1093/bioinformatics/btq461 20709691

[47] Price MN Dehal PS Arkin AP. FastTree 2–approximately maximum-likelihood trees for large alignments. PLoS ONE. (2010) 5:e9490. 10.1371/journal.pone.0009490 20224823 PMC2835736

[48] Katoh K Standley DM. MAFFT multiple sequence alignment software Version 7: improvements in performance and usability. Mol Biol Evol. (2013) 30:772–80. 10.1093/molbev/mst010 23329690 PMC3603318

[49] Stamatakis A. RAxML version 8: a tool for phylogenetic analysis and post-analysis of large phylogenies. Bioinformatics. (2014) 30:1312–3. 10.1093/bioinformatics/btu033 24451623 PMC3998144

[50] Dabdoub SM Fellows ML Paropkari AD Mason MR Huja SS Tsigarida AA. PhyloToAST: bioinformatics tools for species-level analysis and visualization of complex microbial datasets. Sci Rep. (2016) 6:29123. 10.1038/srep29123 27357721 PMC4928119

[51] Letunic I Bork P. Interactive Tree Of Life v2: online annotation and display of phylogenetic trees made easy. Nucleic Acids Res. (2011) 39:W475–8. 10.1093/nar/gkr201 21470960 PMC3125724

[52] Letunic I Bork P. Interactive tree of life (iTOL) v3: an online tool for the display and annotation of phylogenetic and other trees. Nucleic Acids Res. (2016) 44:W242–5. 10.1093/nar/gkw290 27095192 PMC4987883

[53] McMurdie PJ Holmes S. Phyloseq: an R package for reproducible interactive analysis and graphics of microbiome census data. PLoS ONE. (2013) 8:e61217. 10.1371/journal.pone.0061217 23630581 PMC3632530

[54] Oksanen J Blanchet FG Friendly M Kindt R Legendre P McGlinn D. Vegan: Community Ecology Package. (2.5-5 ed.) San Francisco, CA (2019).

[55] Team RC. R: A Language and Environment for Statistical Computing. Vienna: R Foundation for Statistical Computing (2018).

[56] Lozupone C Lladser ME Knights D Stombaugh J Knight R. UniFrac: an effective distance metric for microbial community comparison. ISME J. (2011) 5:169–72. 10.1038/ismej.2010.133 20827291 PMC3105689

[57] Wickham H. ggplot2: Elegant Graphics for Data Analysis. New York, NY: Springer-Verlag (2009). 10.1007/978-0-387-98141-3

[58] Jones RM. The influence of the gut microbiota on host physiology: in pursuit of mechanisms. Yale J Biol Med. (2016) 89:285–97. 27698613 PMC5045138

[59] Larsen OFA Claassen E. The mechanistic link between health and gut microbiota diversity. Sci Rep. (2018) 8:2183. 10.1038/s41598-018-20141-6 29391457 PMC5794854

[60] Rinninella E Raoul P Cintoni M Franceschi F Miggiano GAD Gasbarrini A. What is the healthy gut microbiota composition? A changing ecosystem across age, environment, diet, and diseases. Microorganisms. (2019) 7:14. 10.3390/microorganisms7010014 30634578 PMC6351938

[61] Matsuki T Tajima S Hara T Yahagi K Ogawa E Kodama H. Infant formula with galacto-oligosaccharides (OM55N) stimulates the growth of indigenous bifidobacteria in healthy term infants. Benef Microb. (2016) 7:453–61. 10.3920/BM2015.0168 27120106

[62] So D Whelan K Rossi M Morrison M Holtmann G Kelly JT. Dietary fiber intervention on gut microbiota composition in healthy adults: a systematic review and meta-analysis. Am J Clin Nutr. (2018) 107:965–83. 10.1093/ajcn/nqy041 29757343

[63] Cheng W Lu J Li B Lin W Zhang Z Wei X. Effect of functional oligosaccharides and ordinary dietary fiber on intestinal microbiota diversity. Front Microbiol. (2017) 8:1750. 10.3389/fmicb.2017.01750 28979240 PMC5611707

[64] Szklany K Wopereis H de Waard C van Wageningen T An R van Limpt K. Supplementation of dietary non-digestible oligosaccharides from birth onwards improve social and reduce anxiety-like behaviour in male BALB/c mice. Nutr Neurosci. (2019) 23:896–910. 10.1080/1028415X.2019.1576362 30871432

[65] Ho NT Li F Lee-Sarwar KA Tun HM Brown BP Pannaraj PS. Meta-analysis of effects of exclusive breastfeeding on infant gut microbiota across populations. Nat Commun. (2018) 9:4169. 10.1038/s41467-018-06473-x 30301893 PMC6177445

[66] Backhed F Ley RE Sonnenburg JL Peterson DA Gordon JI. Host-bacterial mutualism in the human intestine. Science. (2005) 307:1915–20. 10.1126/science.1104816 15790844

[67] Ley RE Hamady M Lozupone C Turnbaugh PJ Ramey RR Bircher JS. Evolution of mammals and their gut microbes. Science. (2008) 320:1647–51. 10.1126/science.1155725 18497261 PMC2649005

[68] Van den Abbeele P Verstraete W El Aidy S Geirnaert A Van de Wiele T. Prebiotics, faecal transplants and microbial network units to stimulate biodiversity of the human gut microbiome. Microb Biotechnol. (2013) 6:335–40. 10.1111/1751-7915.12049 23594389 PMC3917468

[69] Ríos-Covián D Ruas-Madiedo P Margolles A Gueimonde M de los Reyes-Gavilán CG Salazar N. Intestinal short chain fatty acids and their link with diet and human health. Front Microbiol. (2016) 7:185. 10.3389/fmicb.2016.00185 26925050 PMC4756104

[70] Rios-Covian D Gueimonde M Duncan SH Flint HJ de los Reyes-Gavilan CG. Enhanced butyrate formation by cross-feeding between *Faecalibacterium prausnitzii* and *Bifidobacterium adolescentis*. FEMS Microbiol Lett. (2015) 362:fnv176. 10.1093/femsle/fnv176 26420851

[71] Leclaire C Lecointe K Gunning PA Tribolo S Kavanaugh DW Wittmann A. Molecular basis for intestinal mucin recognition by galectin-3 and C-type lectins. FASEB J. (2018) 32:3301–20. 10.1096/fj.201700619R 29401627 PMC5976236

[72] Fouladi F Glenny EM Bulik-Sullivan EC Tsilimigras MCB Sioda M Thomas SA. Sequence variant analysis reveals poor correlations in microbial taxonomic abundance between humans and mice after gnotobiotic transfer. ISME J. (2020) 14:1809–20. 10.1038/s41396-020-0645-z 32313261 PMC7305193

[73] Davis LM Martinez I Walter J Hutkins R. A dose dependent impact of prebiotic galactooligosaccharides on the intestinal microbiota of healthy adults. Int J Food Microbiol. (2010) 144:285–92. 10.1016/j.ijfoodmicro.2010.10.007 21059476

[74] Liu F Li P Chen M Luo Y Prabhakar M Zheng H. Fructooligosaccharide (FOS) and Galactooligosaccharide (GOS) increase bifidobacterium but reduce butyrate producing bacteria with adverse glycemic metabolism in healthy young population. Sci Rep. (2017) 7:11789. 10.1038/s41598-017-10722-2 28924143 PMC5603605

[75] Reagan-Shaw S Nihal M Ahmad N. Dose translation from animal to human studies revisited. FASEB J. (2008) 22:659–61. 10.1096/fj.07-9574LSF 17942826

[76] EFSA Panel on Dietetic Products Nutrition and Allergies (NDA). Safety of 2′-O-fucosyllactose as a novel food ingredient pursuant to Regulation (EC) No 258/97. EFSA J. (2015) 13:4184. 10.2903/j.efsa.2015.4184

[77] Balogh R Jankovics P Béni S. Qualitative and quantitative analysis of N-acetyllactosamine and lacto-N-biose, the two major building blocks of human milk oligosaccharides in human milk samples by high-performance liquid chromatography–tandem mass spectrometry using a porous graphitic carbon column. J Chromatograp A. (2015) 1422:140–6. 10.1016/j.chroma.2015.10.006 26477523

[78] Dewey KG Lönnerdal B. Milk and nutrient intake of breast-fed infants from 1 to 6 months: relation to growth and fatness. J Pediatric Gastroenterol Nutrit. (1983) 2:497–506. 10.1097/00005176-198302030-00018 6620057

[79] Thompson AL Monteagudo-Mera A Cadenas MB Lampl ML Azcarate-Peril MA. Milk- and solid-feeding practices and daycare attendance are associated with differences in bacterial diversity, predominant communities, and metabolic and immune function of the infant gut microbiome. Front Cell Infect Microbiol. (2015) 5:3. 10.3389/fcimb.2015.00003 25705611 PMC4318912

[80] Marriage BJ Buck RH Goehring KC Oliver JS Williams JA. Infants fed a lower calorie formula with 2′FL show growth and 2′FL uptake like breast-fed infants. J Pediatr Gastroenterol Nutr. (2015) 61:649–58. 10.1097/MPG.0000000000000889 26154029 PMC4645963

