# Supplementary figures and images for: Safety and Modulatory Effects of Humanized Galacto-Oligosaccharides on the Gut Microbiome
Source: Front Nutr. 2021 Apr 7;8:640100. doi: 10.3389/fnut.2021.640100 (PMC8058378; doi:10.3389/fnut.2021.640100)

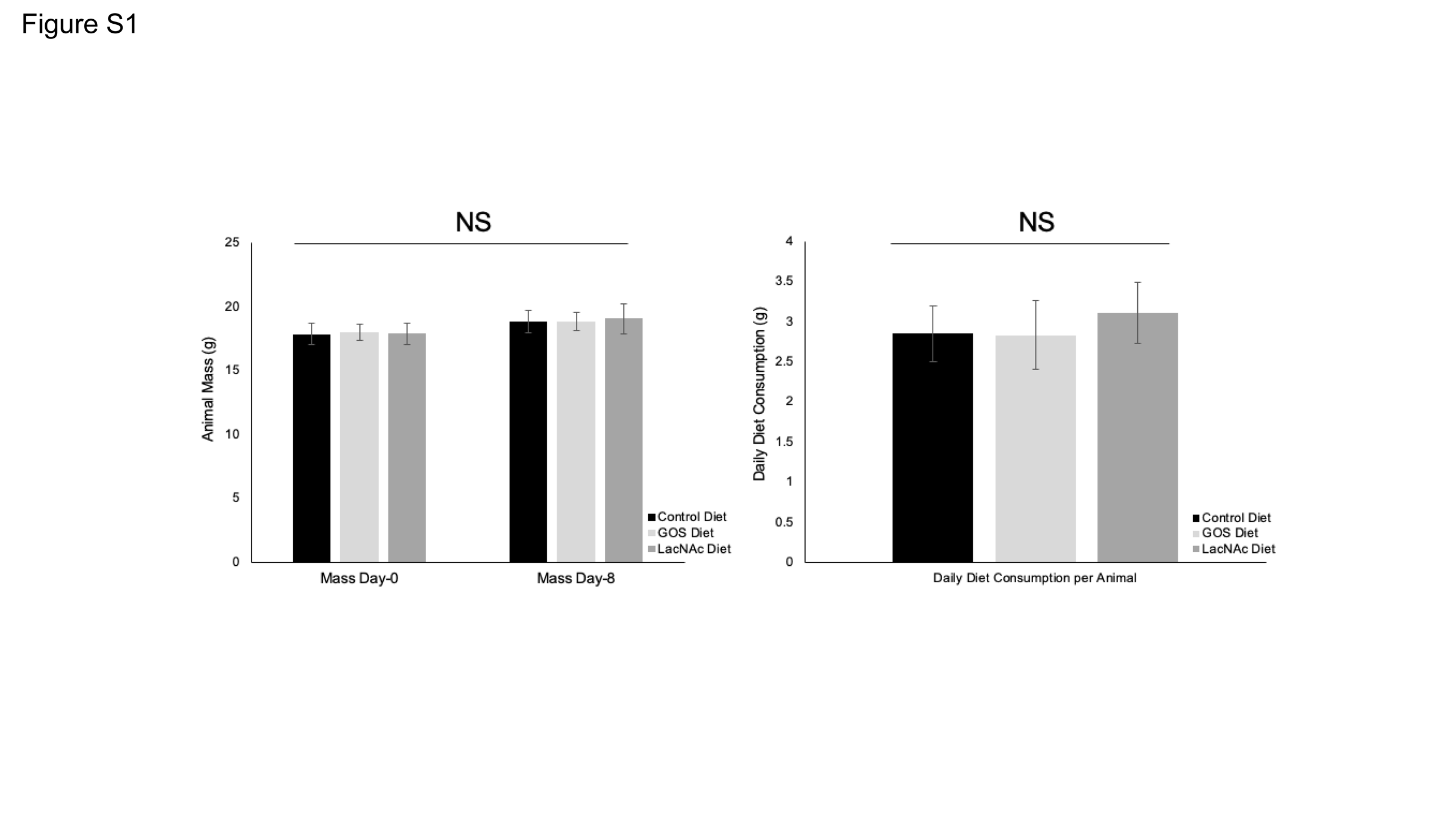

Supplement: Supplementary Figure 1 — Bodyweight and daily dietary consumption datasets are included to show that animal growth and intake were not inhibited by the introduction of either experimental diet (GOS and hGOS). [file Image_1.TIF]

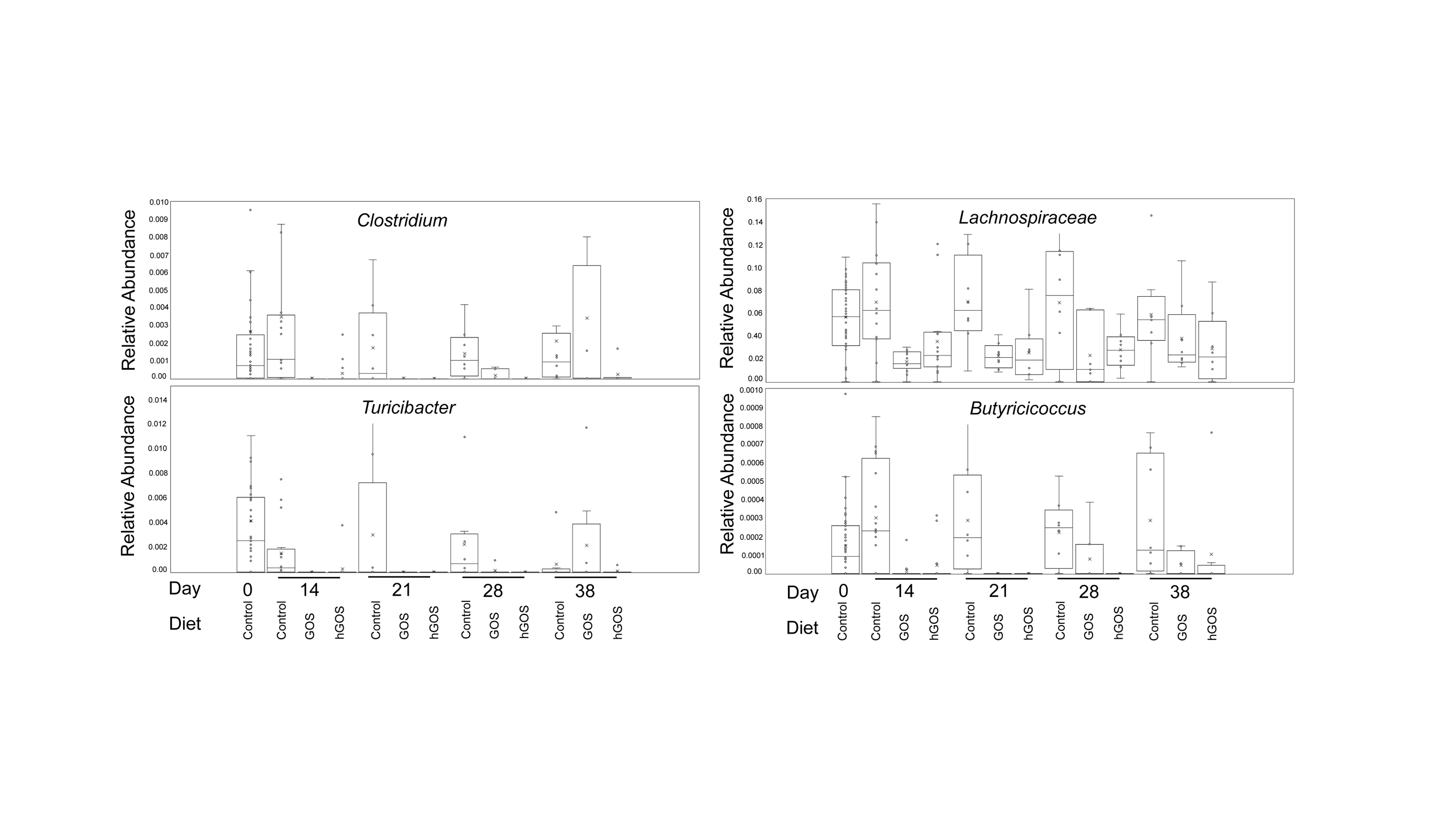

Supplement: Supplementary Figure 2 — Bacterial taxa shown to have a significantly reduced relative abundance in the presence of hGOS. [file Image_2.TIF]
