# Supplementary material for: Safety and Modulatory Effects of Humanized Galacto-Oligosaccharides on the Gut Microbiome
Source: Front Nutr. 2021 Apr 7;8:640100. doi: 10.3389/fnut.2021.640100 (PMC8058378; doi:10.3389/fnut.2021.640100)
Supplement: Supplementary Table 1 — Composition of diets used in the animal study. [file Table_1.DOCX]

**Table S1. Composition of diets used in the animal study.**

| **Product Number** | **D17127301** | | **D17121302** | | **D18121401** | |
| --- | --- | --- | --- | --- | --- | --- |
|  | Control Diet | | GOS Diet | | hGOS Diet | |
|  | gm% | kcal% | gm% | kcal% | gm% | kcal% |
| Protein | 19.3 | 20 | 19.8 | 20 | 19.8 | 20 |
| Carbohydrate | 62.8 | 65 | 61.8 | 62 | 61.8 | 62 |
| Fat | 6.7 | 15 | 6.8 | 15 | 6.8 | 15 |
| kcal/gm | 3.88 |  | 3.98 |  | 3.98 |  |
|  |  |  |  |  |  |  |
| **Ingredient** | **gm** | **kcal** | **gm** | **kcal** | **gm** | **kcal** |
| Casein | 200 | 800 | 200 | 800 | 200 | 800 |
| L-Cystine | 3 | 12 | 3 | 12 | 3 | 12 |
|  |  |  |  |  |  |  |
| Corn Starch | 433.2 | 1732.8 | 413.5 | 1654 | 413.5 | 1654 |
| Maltodextrin 10 | 110 | 440 | 110 | 440 | 110 | 440 |
| Dextrose | 100 | 400 | 100 | 400 | 100 | 400 |
| Lactose | 7.2 | 29 | 0 | 0 | 0 | 0 |
|  |  |  |  |  |  |  |
| Cellulose, BW200 | 71.8 | 0 | 0 | 0 | 0 | 0 |
| Galacto-oligosaccharides (GOS) | 0 | 0 | 71.8 | 108 | 0 | 0 |
| LacNAc Enriched GOS (hGOS) | 0 | 0 | 0 | 0 | 71.8 | 108 |
|  |  |  |  |  |  |  |
| Soybean Oil | 70 | 630 | 70 | 630 | 70 | 630 |
|  |  |  |  |  |  |  |
| Mineral Mix S10026 | 10 | 0 | 10 | 0 | 10 | 0 |
| Dicalcium Phosphate | 13 | 0 | 13 | 0 | 13 | 0 |
| Calcium Carbonate | 5.5 | 0 | 5.5 | 0 | 5.5 | 0 |
| Potassium Citrate, 1 H_2_O | 16.5 | 0 | 16.5 | 0 | 16.5 | 0 |
|  |  |  |  |  |  |  |
| Vitamin Mix V10001 | 10 | 40 | 10 | 40 | 10 | 40 |
| Choline Bitartrate | 2 | 0 | 2 | 0 | 2 | 0 |
|  |  |  |  |  |  |  |
| Red Dye #40, FD&C | 0 | 0 | 0.05 | 0 | 0.025 | 0 |
| Blue Dye #1, FD&C | 0.05 | 0 | 0 | 0 | 0.025 | 0 |
|  |  |  |  |  |  |  |
| **Total** | **1052.25** | **4085** | **1025.35** | **4085** | **1025.35** | **4085** |
|  |  |  |  |  |  |  |
